# *Fusobacterium nucleatum* promotes inflammatory and anti-apoptotic responses in colorectal cancer cells via ADP-heptose release and ALPK1/TIFA axis activation

**DOI:** 10.1080/19490976.2023.2295384

**Published:** 2023-12-21

**Authors:** Camille Martin-Gallausiaux, Laurène Salesse, Diego Garcia-Weber, Ludovica Marinelli, Fabienne Beguet-Crespel, Vincent Brochard, Camille Le Gléau, Alexandre Jamet, Joël Doré, Hervé M. Blottière, Cécile Arrieumerlou, Nicolas Lapaque

**Affiliations:** a Université Paris-Saclay, INRAE, AgroParisTech, Micalis Institute, Jouy-en-Josas, France; b Université Paris Cité, CNRS, INSERM, Institut Cochin, Paris, France; c Université Paris-Saclay, INRAE, Metagenopolis, Jouy-en-Josas, France

**Keywords:** Fusobacterium, ALPK1, colorectal cancer, NF–κB

## Abstract

The anaerobic bacterium *Fusobacterium nucleatum* is significantly associated with human colorectal cancer (CRC) and is considered a significant contributor to the disease. The mechanisms underlying the promotion of intestinal tumor formation by *F. nucleatum* have only been partially uncovered. Here, we showed that *F. nucleatum* releases a metabolite into the microenvironment that strongly activates NF-κB in intestinal epithelial cells via the ALPK1/TIFA/TRAF6 pathway. Furthermore, we showed that the released molecule had the biological characteristics of ADP-heptose. We observed that *F. nucleatum* induction of this pathway increased the expression of the inflammatory cytokine IL-8 and two anti-apoptotic genes known to be implicated in CRC, *BIRC3* and *TNFAIP3*. Finally, it promoted the survival of CRC cells and reduced 5-fluorouracil chemosensitivity *in vitro*. Taken together, our results emphasize the importance of the ALPK1/TIFA pathway in *Fusobacterium* induced-CRC pathogenesis, and identify the role of ADP-H in this process.

## Introduction

Colorectal cancer (CRC) is one of the most common cancers, ranked third in incidence (1.8 million new cases/year) and is the second leading cause of cancer-related mortality (881000 deaths).^1^ Although the etiology of CRC remains unclear, intestinal inflammation and various genetic factors contribute to CRC development. However, the relatively low heritability of CRC emphasizes the importance of environmental factors in disease development. Such factors encompass lifestyle changes that have occurred in developed societies over the last century, potentially affecting intestinal microbiota composition (antibiotics, smoking, diet low in fibers, and consumption of highly processed foods).^2^

Sequencing approaches have been applied to explore microbiome profiles and unveiling distinct taxonomic bacterial composition referred to as dysbiosis in patients with CRC when compared to healthy individuals.^3,4^ Furthermore, recent research has evidenced the role of several intestinal bacteria in the development and severity of numerous cancers and also in influencing the effectiveness of cancer therapies.^3,4^ The impact of *Porphyromonas, Escherichia, Bacteroides, Streptococcus* and *Fusobacterium* has been particularly investigated in CRC. These species are often enriched in tumor tissues compared to healthy adjacent mucosa.^5–8^ Among these species, the gram-negative anaerobic bacterium *Fusobacterium nucleatum* stands out as the most prevalent bacterium associated with CRC^9,10^ across different stages and subgroups of the disease.^3,4,8,9,11–15^ Numerous studies have consistently reported an enrichment *F. nucleatum* in CRC samples from populations of diverse geographical origins, including Europe, Asia and America highlighting a worldwide relevance.^3,4,8,12,15–17^ Moreover, *F. nucleatum* presence in CRC patients is associated with poor patient prognosis and resistance to chemotherapy.^18,19^ In recent years, the impact of *F. nucleatum* on CRC has been extensively studied using cellular and animal models. Mechanistically, the *F. nucleatum* effector, *Fusobacterium* adhesin protein 2 (Fap2), has been reported to bind host epithelial cells by interacting with the tumor-specific sugar residue Gal-GalNAc, facilitating *Fusobacterium* localization and enrichment in CRC.^20^ In addition, a study has demonstrated that the effector *Fusobacterium* adhesin A (FadA), expressed at the membrane of *F. nucleatum*, activates the Wnt/β-catenin signaling pathway leading to the induction of oncogenic and inflammatory responses.^21^ FadA has also been reported to bind to E-cadherin and to induce DNA damage such as DNA Double-Strand breaks (DSBs), resulting in chromosomal instability and cancer development.^22^ Finally, *F. nucleatum* modulates the tumor-immune microenvironment by inhibiting natural killer cell (NK) cytotoxicity through Fap2, which binds to the human immune inhibitory receptor T-cell immunoglobulin and ITIM domain (TIGIT).^23^ Interestingly, it has been demonstrated that *Fusobacterium* promotes the proliferation and reduces the chemosensitivity of CRC cells. This occurs through the activation of the transcription factor NF-κB, by *Fusobacterium* lipopolysaccharide (LPS), leading to the dysregulation of the autophagy and the anti-apoptotic pathways.^18,19,24^ NF-κB contributes to intestinal homeostasis and is induced by microbial ligands recognized by Pattern recognition receptors (PRRs). Indeed, dysregulation of the PRR-dependent NF-κB signaling pathway has been linked to chronic inflammation, as well as dysfunction in barrier and tissue repair mechanisms. These dysfunctions often lead to excessive repair responses and cell proliferation, which are involved in various stages of carcinogenicity.^25–27^ Despite being one of the most extensively studied CRC-related bacteria, the mechanisms through which *F. nucleatum* activates NF-κB and consequently drives CRC pathogenesis have not been fully characterized.

Recently, Alpha kinase 1 (ALPK1), a new PRR that senses ADP-heptose (ADP-H) produced by pathogenic and commensal gram-negative bacteria, has been described.^28–31^ The binding of ADP-H to ALPK1 induces TIFA phosphorylation and subsequent NF-κB activation, which has been suggested to play a role in the pro-inflammatory response against pathogens.^29,32,33^ A recent study showed that ALPK1 induces ICAM-1 expression upon *F. nucleatum* exposure, increasing CRC cell adhesion to endothelial cells.^34^ Interestingly, ALPK1 regulates intestinal homeostasis in a mouse model of colitis^35^ and single-nucleotide polymorphisms (SNPs) in this gene are associated with a range of chronic inflammatory diseases and various cancers, including CRC.^36–40^ A study has shown that TIFA is involved in the Aurora A- and NF-κB-dependent expression of anti-apoptotic factors involved in leukemic cell growth and chemoresistance in acute myeloid leukemia.^41^ More importantly, the activation of the ALPK1-TIFA-NF-κB axis is directly linked to pro-oncogenic processes, promoting replication stress and DNA damage in gastric cells.^42^ Collectively, these studies suggest that ALPK1 play a role in promoting carcinogenesis; however, the specific pro-oncogenic processes involved and their connection with gut bacteria have not been definitively established.

In the present study, we showed that *F. nucleatum* releases molecules into its microenvironment, which activate NF-κB in intestinal epithelial cells (IECs) independently of TLRs and Nucleotide-binding oligomerization domain-containing protein (NOD) receptors, and through the ALPK1/TIFA pathway. This ALPK1/TIFA-dependent activation of the NF-κB phenotype was conserved among all fusobacterial species tested and acted synergistically with the butyrate produced by these bacteria. We investigated the impact of ALPK1/TIFA activation by *F. nucleatum* supernatant on HT-29 CRC cells and observed that induction of this pathway increased the expression of two anti-apoptotic genes and reduced the chemosensitivity of CRC cell to 5-fluorouracil. Taken together, our study provides novel evidence suggesting that the ALPK1/TIFA pathway is associated with the remote effect of *Fusobacterium* on pro-oncogenic processes.

## Results

### Fusobacterium nucleatum releases molecules that induce NF-κB activity in IECs through ALPK1/TIFA/TRAF6 pathway, independently of TLRs and NODs

**Figure 1.**
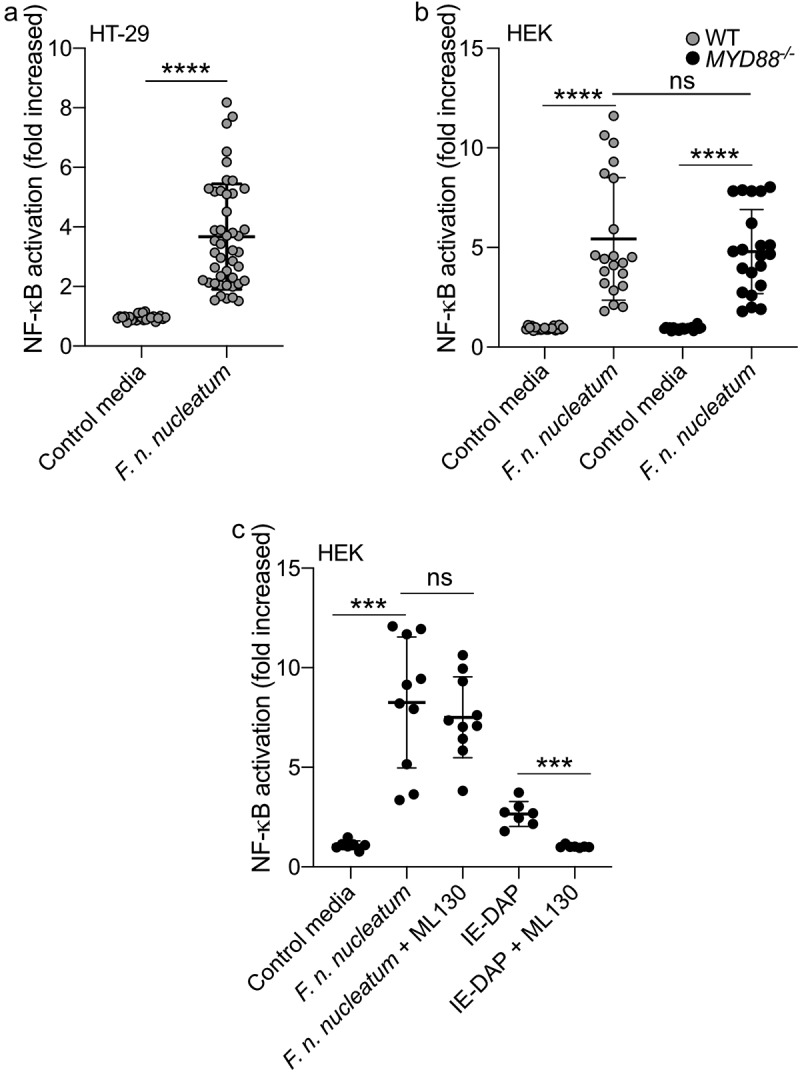
*F. nucleatum* activates NF-κB in HT-29 independently of MyD88 and NOD1. (a) Average NF-κB activity in HT-29-NFκB reporter system (10% vol/vol), induced by *F. nucleatum* supernatant or control media for 24 h. (b) HEK-NFκB reporter (WT, black bars) and deleted for MYD88 (MYD88^−/−^, gray bars) cells were incubated with *F. nucleatum* supernatant or control media for 24 h. (c) Treatment of HEK-NFκB reporter cells deleted for MYD88^−/−^ with control media, *F. nucleatum* supernatant or the NOD1 ligand (IE-DAP) for 24 h in presence or absence of a NOD1 inhibitor (ML130). NF-κB activation was measured by SEAP secretion and expressed as mean ± SD fold change toward unstimulated cells. Data represent ≥ 3 independent experiments performed in technical duplicate or triplicate. Data analysis: Mann-Whitney test was used, ****P < .0001; ***P < .001; **P < .01; *P < ,05; *P* < .05 was considered as not significant (ns).

Previous studies have established that *F. nucleatum* is a potent activator of the pro-inflammatory transcription factor NF-κB in a wide range of cell-lines.^34,43^ Exposure to *F. nucleatum* leads to the activation of TLRs, NODs, and ALPK1, resulting in NF-κB responses in various cell types; however, the impact of the molecules released in the microenvironment by the bacterium has not been characterized.^34,44–46^ We used culture supernatants from *F. nucleatum* subsp. *nucleatum* (DMS15643) and found that this bacterium released molecules capable of activating NF-κB in a reporter system expressed in the intestinal epithelial cell (IEC) line HT-29 (HT-29-NF-κB cells) (Figure 1a). Additionally, *F. nucleatum* supernatant activated NF-κB in HEK cells bearing the same reporter system (HEK-NF-κB). Importantly, this HEK cell line lacks expression of TLR2 and TLR4, which are linked to *F. nucleatum*’s major activity in CRC (Figure 1b).^18,19,24,34,44,45^ To investigate whether the remaining TLRs expressed in HEK-NF-κB were responsible for the NF-κB response to *F. nucleatum* supernatants, we deleted the gene encoding the adaptor protein MYD88 (*MYD88*^*-/-*^), a key protein in TLR signaling pathways. Interestingly, when incubated with *F. nucleatum* supernatants, both HEK-NF-κB WT and *MYD88*^*-/-*^ cells exhibited similar NF-κB activity, indicating that the supernatant induced NF-κB activation via a MYD88-independent pathway (Figure 1b). Altogether, our results strongly demonstrate that *F. nucleatum* molecules released into the microenvironment activate NF-κB independently of MYD88 and TLRs.

NOD1 and NOD2-dependent signaling pathways are reported to induce NF-κB activation in response to fusobacterial infections.^47^ We excluded the involvement of NOD2 in *F. nucleatum*-dependent NF-κB activation, as this receptor is not expressed in HEK cells.^48^ To investigate the role of NOD1, we treated *MYD88*^−/−^ HEK cells with a specific NOD1-inhibitor (ML130). As expected, ML130 treatment completely abolished the activation of NF-κB by the purified NOD1 ligand IE-DAP. However, it did not affect NF-κB activation by *F. nucleatum* supernatant, indicating that NOD1 was not involved in the *F. nucleatum*-dependent activation of NF-κB (Figure 1c).

**Figure 2.**
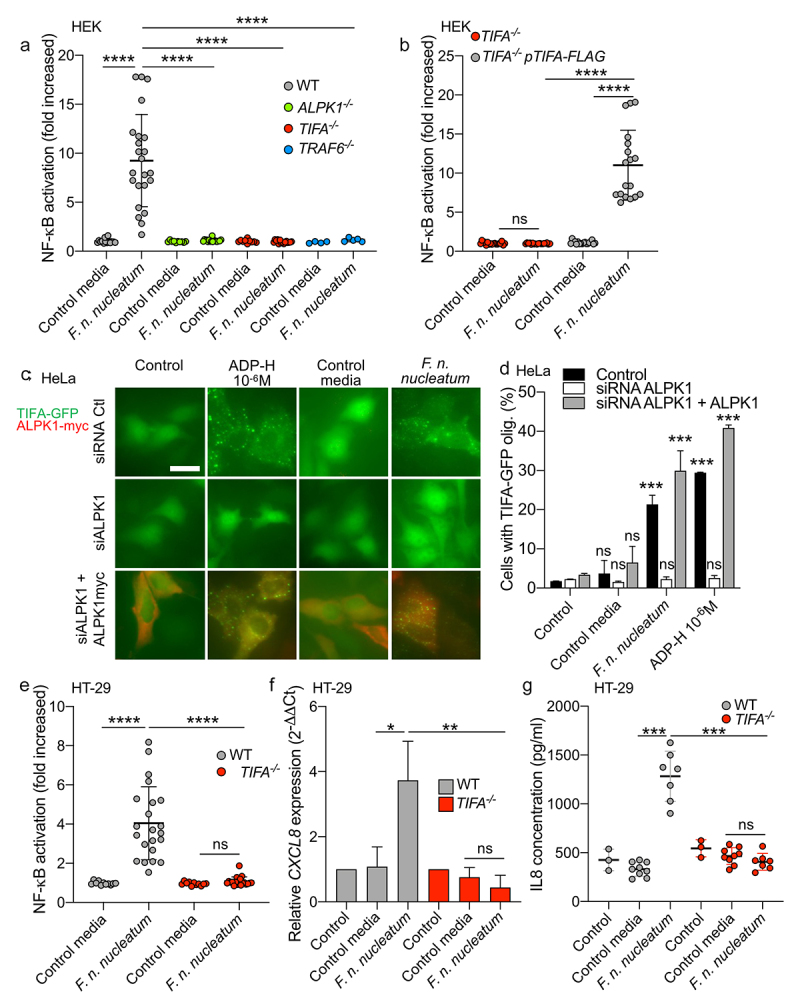
*F. nucleatum* supernatant activates NF-κB via ALPK1, TIFA and TRAF6. (a) WT (gray dots), *ALPK1*^−/−^ (green dots), *TIFA*^−/−^ (red dots) and *TRAF6*^−/−^ (bleu dots) HEK NF-κB-reporter cells were stimulated with *F. nucleatum* supernatant or control media for 24 h. NF-κB activation was measured by SEAP secretion and expressed as mean ± SD fold change toward unstimulated cells. (b) *TIFA*^−/−^ HEK NF-κB-reporter cells transfected (gray dots) or not (red dots) with pTIFA-FLAG and stimulated with *F. nucleatum* supernatant or control media for 24 h. NF-κB activation was measured by SEAP secretion and expressed as mean ± SD fold change toward unstimulated cells. (c,d) TIFA-GFP HeLa cells were treated with control siRNA or ALPK1 specific siRNA prior to transfection with empty pCMV or pCMV-ALPK1 and were left unstimulated (control) or stimulated with ADP-H (10^−6^ M), *F. nucleatum* supernatant or control media for 24 h. Representative pictures of cells with TIFAsomes after 30 min of stimulation are in C (scale bar: 20 μm) and the graph showing the TIFAsomes quantification per cell in each condition is in d (control siRNA in black bars, siRNA ALPK1 in white bars and siRNA ALPK1 + p*ALPK1* in gray bars). (e) WT (gray dots) and *TIFA*^−/−^ (red dots) HT-29 NF-κB reporter cells were stimulated with *F. nucleatum* supernatant or control media for 24 h. NF-κB activation was measured by SEAP secretion and expressed as mean ± SD fold change toward unstimulated cells. (f) *CXCL8* relative expression to GAPDH in WT (black bars) and TIFA^−/−^ (red bars) in HT29 stimulated with stimulated with ADP-H (10^−6^M), *F. nucleatum* supernatant or control media for 6 h expressed as 2^−ΔΔCt^ toward unstimulated cells. g. IL-8 ELISA performed on WT (gray dots) and TIFA^−/−^ (red dots) cells unstimulated (control) or treated with *F. nucleatum* supernatant or control media for 24 h. IL8 concentration was expressed in pg/ml. Data represent ≥ 3 independent experiments performed in duplicate or triplicate. Statistical significance was assessed using Mann-Whitney test (B, E-G) or one-way ANOVA followed by Tukey’s multiple comparisons test (A and D, for D, the samples were compared to their respective control transfected with control siRNA, siRNA ALPK1, siRNA ALPK1 + ALPK1). ****P < .0001; ***P < .001; **P < .01; *P < .05; *P* < .05 was considered as not significant (ns).

ALPK1 serves as an important cytosolic receptor in the innate immune response to gram-negative bacteria. Previous studies have demonstrated its activation upon exposure to *F. nucleatum*, ^34^ which lead us to hypothesize that ALPK1 might be involved in the NF-κB activation induced by *F. nucleatum* supernatant. ALPK1 senses intracellular ADP-H, leading to TIFA phosphorylation and consequently TIFA and TRAF6 oligomerization, resulting in the downstream activation of NF-κB.^28,30^ To validate our hypothesis, we assessed NF-κB activation by *F. nucleatum* supernatant in WT, ALPK1^−/−^, TIFA^−/−^ and TRAF6^−/−^ HEK cells. Our results demonstrated that the NF-κB response was dependent on the presence of ALPK1, TIFA, and TRAF6 (Figure 2a). Furthermore, we confirmed the critical role of this pathway by ectopically expressing TIFA in HEK TIFA^−/−^ cells, which successfully restored NF-κB activation by *F. nucleatum* supernatant (Figure 2b).

**Figure 3.**
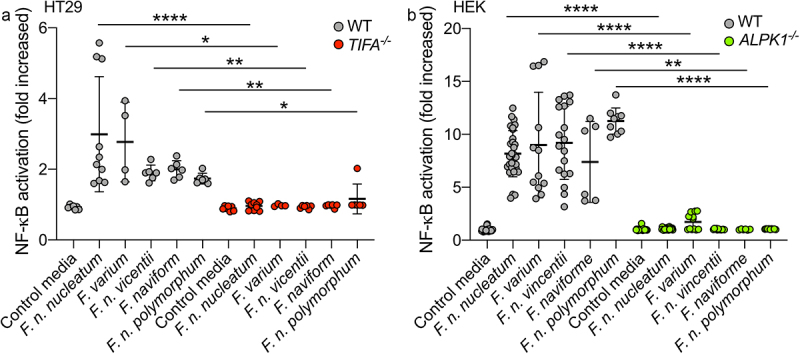
*Fusobacterium* species activate NF-κB *via* the ALPK1-TIFA pathway. (a) WT (gray dots) and TIFA^−/−^ (red dots) HT-29 NF-κB-reporter cells were stimulated with supernatants derived from different *Fusobacterium* spp. Or control media for 24 h. (b) WT (gray dots) and ALPK1^−/−^ (green dots) HEK NF-κB-reporter cells were incubated with supernatants derived from different *Fusobacterium* spp. Or control media for 24 h. NF-κB activation was measured by SEAP secretion and expressed as mean ± SD fold change toward unstimulated cells. Data represent ≥ 3 independent experiments performed in triplicate. Data analysis: Mann-Whitney test was used, ****P < ,0001; ***P < ,001; **P < ,01; *P < ,05; *P* < 0.05 was considered as not significant (ns).

Upon stimulation of ALPK1 by its ligand, TIFA is phosphorylated, leading to the oligomerization of TIFA proteins into large structures called TIFAsomes^28,30^. Therefore, we assessed the impact of *F. nucleatum* supernatant on TIFAsome formation and investigated the role of ALPK1 in this process. To quantify TIFAsome formation, we used cells stably expressing GFP-tagged TIFA (TIFA-GFP) and transfected them with control or ALPK1-targeting siRNAs for gene silencing. In this experimental setup, we observed that both the bacterial supernatant and synthetic ADP-H induced TIFAsome formation, and that this phenotype was abolished in ALPK1-depleted cells. Furthermore, TIFAsome formation was restored in ALPK1-depleted cells upon rescue with an ectopically transfection of an ALPK1 cDNA construct (Figure 2c, d). To establish the relevance of these findings in intestinal epithelial cells, we showed that knocking out TIFA in HT-29 cells resulted in the abrogation of NF-κB activation by *F. nucleatum* supernatant and the downstream induction of IL-8 (encoded by *CXCL8*) at both the transcriptional and protein levels (Figure 2e, f). Moreover, ADP-H and *F. nucleatum* activated both NF-κB response and *CXCL8* expression in the HCT116 cell-line, suggesting that the ALPK1 pathway is functional in other IECs (Supplementary Figure S1). Interestingly, we observed that the activation of the ALPK1/TIFA axis was not specific to *F. nucleatum* subsp. *nucleatum*, as all the fusobacterial species and strains tested exhibited similar ALPK1 and TIFA-dependent activation of NF-κB (Figure 3a, b). Taken together, our results provide strong support for the role of the ALPK1/TIFA/TRAF6 pathway in the activation of NF-κB by *Fusobacterium* culture supernatants in the intestinal context.

### Fusobacterium nucleatum releases in its microenvironment a NF-κB-activating molecule that has the biological features of the ALPK1 ligand, ADP-heptose

**Figure 4.**
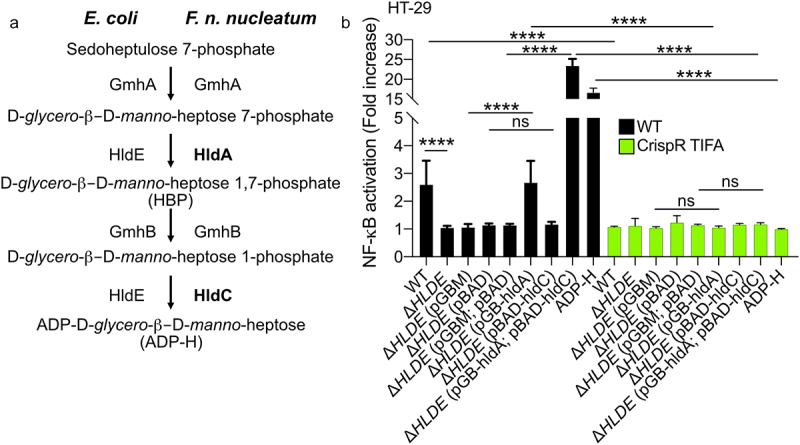
*F. nucleatum* enzymes HldA (Fn1786) and HldC (Fn0930) from the heptose biosynthesis pathway are functional. (a) Schematic view of the heptose biosynthesis pathway. (b) HT-29-NF-κB reporter WT (black bars) or TIFA^−/−^ (green bars) cells were stimulated for 24 h with lysates from *E. coli*; Δ*hldE*; Δ*hldE* transformed with plasmid controls (pBAD and/or pGB), with *hldA* (pGB-hldA), with *hldC* (pBAD-hldC), with *hldA* (pGB-hldA) and *hldC* (pBAD-hldC) from *F. nucleatum* or ADP-H (10^−6^ M). NF-κB activation was measured by SEAP secretion and expressed as mean (%) ± SD fold change toward supernatant-stimulated cells. Data represent ≥ 3 independent experiments performed in triplicate. Data analysis: one-way ANOVA followed by Tukey’s multiple comparisons test was used, ****P < .0001; ***P < .001; **P < .01; *P < .05; *P* <.05 was considered as not significant (ns).

ADP-H and heptose-1,7-bisphosphate (HBP) are metabolites produced by gram-negative bacteria that trigger the ALPK1/TIFA pathway and consequently activate NF-κB.^28,29^ These molecules are intermediates in the heptose biosynthesis pathway, involved in LPS synthesis.^29^ The KEGG database and genomic analysis revealed the presence of genes encoding enzymes (GmhA, HldA, HldC, and GmhB) involved in the LPS pathway in the genome of *F. nucleatum* (Figure 4a and Supplementary Figure S2). Among these, HldA functions as a heptokinase, while HldC acts as an ADP-transferase enzyme, producing HBP and ADP-H respectively. In *E. coli*, both these enzymatic activities are carried out by the bifunctional enzyme HldE.^29^ We took advantage of an *hldE* deletion mutant of *E. coli* (Δ*hldE*), in which we expressed *F. nucleatum hldA* and *hldC* to determine whether these enzymes were functional. Since ADP-H is not secreted by *E. coli*, bacterial lysates were used. Our results showed that lysates from WT and Δ*hldE E. coli* expressing *hldA* or both *hldA* and *hldC* from *F. nucleatum* potently activated NF-κB in HT-29 cells (Figure 4b), whereas the lysate of Δ*hldE* mutant did not, confirming the functionality of both enzymes. The mechanism was mediated by the ALPK1-TIFA pathway, as shown by the impaired NF-κB activation observed in HT-29 TIFA^−/−^ cells treated with bacterial lysates from WT, Δ*hldE* alone, Δ*hldE* p*hldA*-, and Δ*hldE* p*hldA*/p*hldC*-expressing *E. coli* (Figure 4b). Notably, the expression of both enzymes was required for optimal NF-κB activation, indicating that ADP-H is a more potent activator than HBP. Altogether, these results show that the expression of *F. nucleatum hldA* or both *hldA/hldC* in *E. coli* is sufficient to activate NF-κB in a TIFA-dependent manner (Figure 4b).

**Figure 5.**
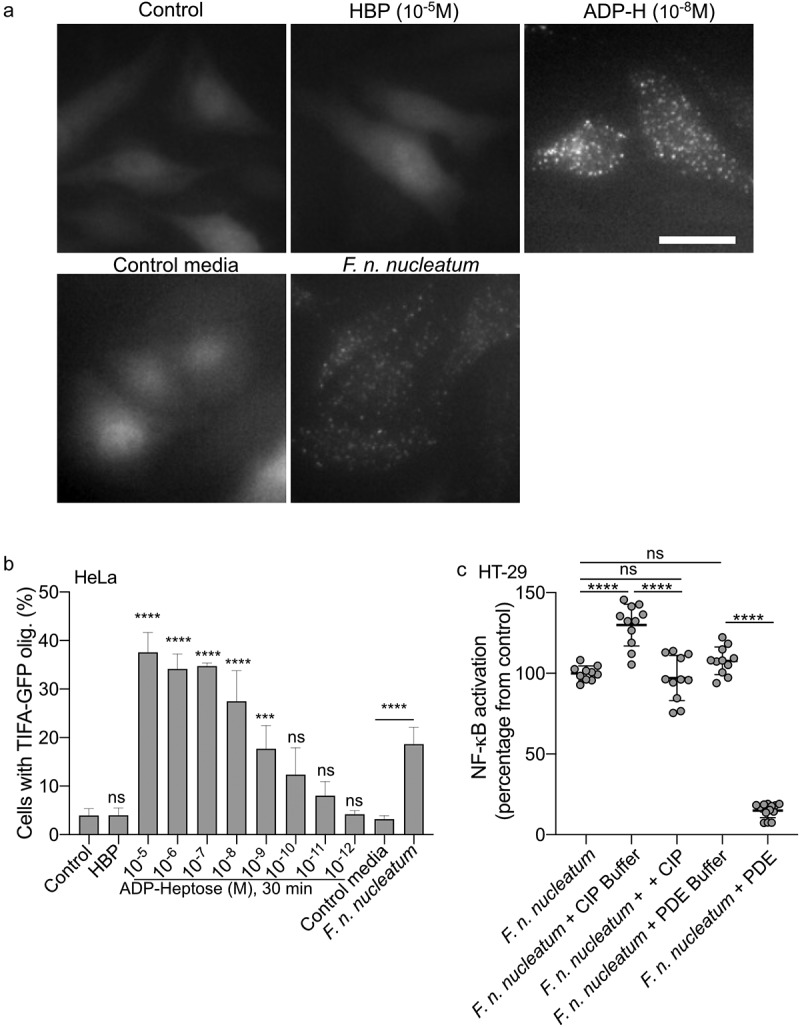
*F. nucleatum* NF-κB activating molecule has the biological features of the ALPK1 ligand ADP-heptose. (a,b) cells were treated with digitonin and non-inoculated control medium, *F. nucleatum* supernatants, HBP (10^−5^M) or ADP-H (10^−8^M). in A: Representative pictures of cells with TIFAsomes at 30 min in TIFA-GFP-expressing HeLa cells (scale bar: 20 μm). In B, quantification of TIFAsomes in each condition as in a from three independent experiments performed in triplicate. (c) *F. nucleatum* supernatant was untreated, treated with calf intestine alkaline phosphatase (CIP), with *C. adamanteus* phosphodiesterase (PDE) or their respective buffer (CIP buffer and PDE buffer) prior to stimulation of HT-29-NF-κB reporter cells for 24 h. NF-κB activation was measured by SEAP secretion and results were expressed as mean change (%) ± SD toward *F. nucleatum* supernatant-stimulated cells. Data represent ≥ 3 independent experiments performed in triplicate. Statistical significance was assessed using one-way ANOVA followed by Tukey’s multiple comparisons test (for B, the samples were compared to their respective control: control for ADP-H and HBP and control media for *F. nucleatum* supernatants). ****P < ,0001; ***P < ,001; **P < ,01; *P < ,05; *P* < 0.05 was considered as not significant (ns).

In contrast to ADP-H, HBP can indirectly and weakly activate the ALPK1/TIFA pathway after undergoing intracellular processing by host adenyltransferases into ADP-H 7-P.^29^ To assess the contribution of HBP or ADP-H in the *F. nucleatum* activation of the ALPK1 pathway, we exploited the fact that, unlike ADP-H, HBP is unable to induce the formation of TIFAsomes within a 30-minutes of treatment.^28^ Within this stimulation period, we observed that supernatants from *F. nucleatum* and synthetic ADP-H induced the formation of TIFAsomes, while HBP failed to do so (Figure 5a, b). The kinetics of TIFAsome formation induced by *F. nucleatum* were consistent with those of ADP-H, but not HBP. ADP-H exhibits partial resistance to calf intestinal alkaline phosphatase (CIP) but is sensitive to phosphodiesterase (PDE) from *C. adamanteus*, whereas HBP is only sensitive to CIP treatment.^49^ To further confirm the role of ADP-H in *F. nucleatum-*induced NF-κB activation and exclude the contribution of HBP, we analyzed the effects of CIP and PDE treatments on bacterial culture supernatants. We showed that the NF-κB-activating molecule secreted by *F. nucleatum* was partially resistant to CIP and entirely sensitive to PDE (Figure 5c). Altogether, our findings support the conclusion that ADP-H, and not HBP, is responsible for the ALPK1-dependent activation of NF-κB by*F. nucleatum* supernatant.

### Release of ADP-heptose and butyrate by fusobacterium synergize the ALPK1-dependentactivation of NF-κB by fusobacterium

**Figure 6.**
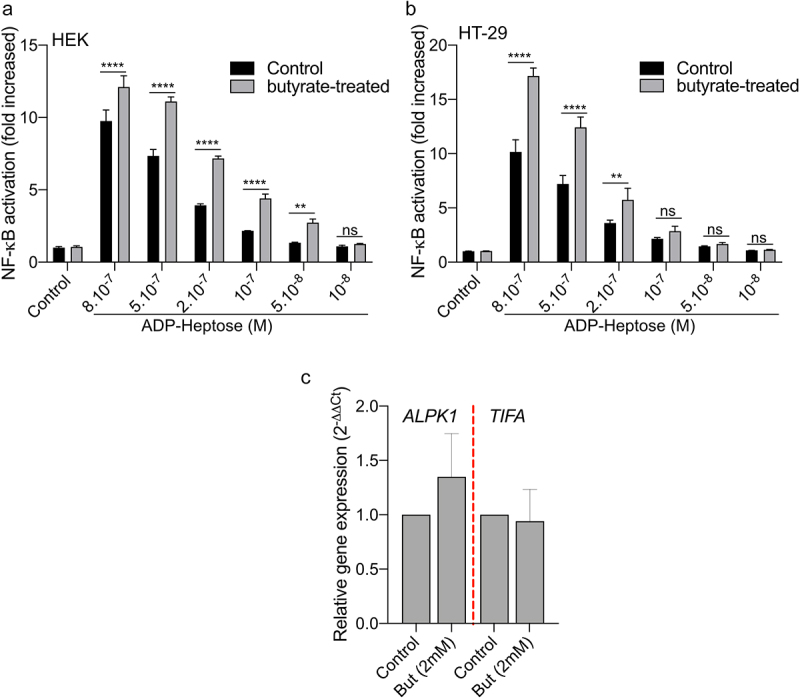
Butyrate synergizes with ADP-H-dependent activation of NF-κB.A-B HEK (a) and HT-29 (b) NF-B-reporter cells were left untreated (control) or stimulated with ADP-H (10^−8^M to 8.10^−7^M) with (gray bars) or without (black bars) butyrate (2 mM). NF-κB activation was measured by SEAP secretion and expressed as the mean ± SD fold change toward unstimulated cells. Data represent three independent experiments performed in triplicate. (c) Real-time qPCR (RT-qPCR) showing *ALPK1* and *TIFA* gene expression in HT-29 cells stimulated with 2 mM butyrate for 6 h. Results were normalized to *GAPDH* and expressed as 2^−ΔΔCt^ toward unstimulated cells. Data represent three independent experiments performed in triplicate. Statistical significance was assessed using two-way ANOVA followed by Tukey’s multiple comparisons test. ****P < ,0001; ***P < ,001; **P < ,01; *P < ,05; *P* < 0.05, was considered as not significant (ns).

*Fusobacterium* species produce short-chain fatty acids, including butyrate, which can have a significant impact on host responses^50–52^(Supplementary Table S1). Butyrate has been reported as a synergistic inducer of NF-κB responses when combined with stimulation of TLRs and NODs.^53–55^ In light of this, we investigated the potential synergy in NF-κB activation between butyrate and ALKP1-dependent signaling. We treated both the HT-29- and HEK-NF-κB reporter systems with butyrate (2 mM) and various concentrations of ADP-H (ranging from 8.10^−7^ to 10^−8^M) (Figure 6a, b). Butyrate alone did not induce NF-κB activation in either of the cell line. However, addition of butyrate (2 mM) to different concentrations of ADP-H led to a significant increase of NF-κB activation, nearly doubling the response compared to ADP-H stimulation alone (Figure 6a, b). It is well established that butyrate has a broad impact on the expression of host genes. We thus analyzed the expression of *ALPK1* and *TIFA* following butyrate stimulation and found that butyrate had no effect on the transcription of these two genes (Figure 6c). Our results thus indicate that butyrate synergize with ADP-H to activate the NF-κB response.

### F. nucleatum supernatant promotes CRC cells proliferation and the expression of anti-apoptotic but not autophagic pro-cancerous genes via the ALPK1/TIFA pathway.

**Figure 7.**
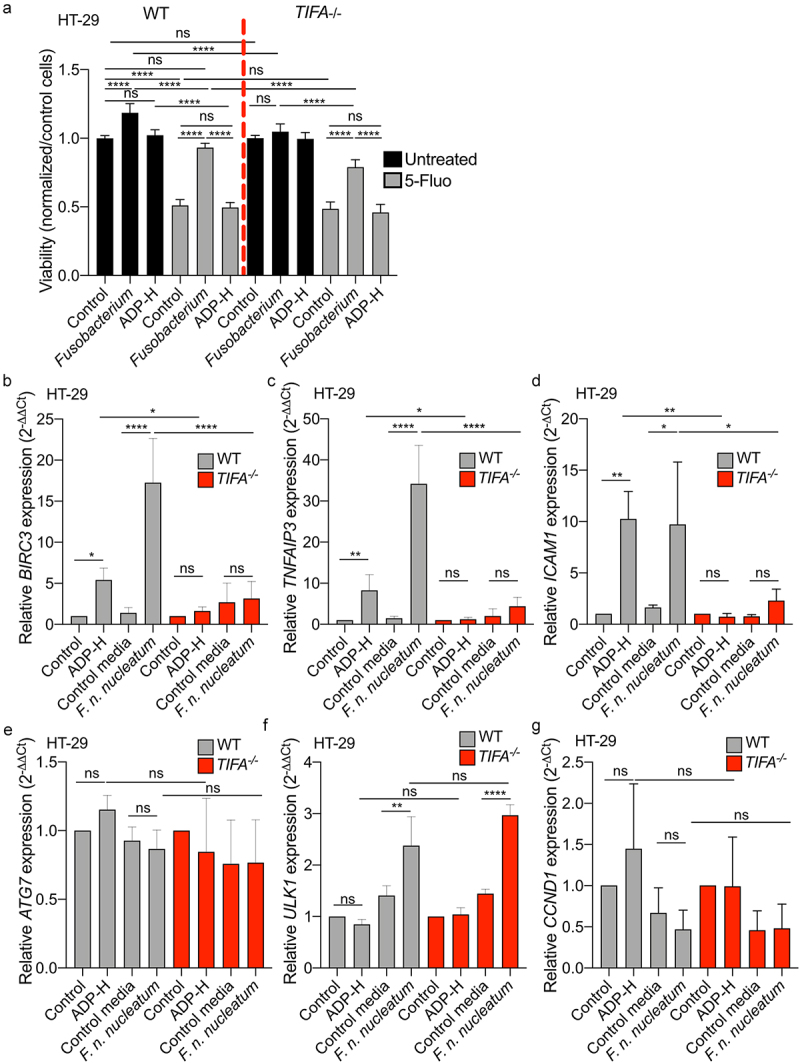
*F. nucleatum* increases HT-29 proliferation and activates anti-apoptotic but not autophagic genes expression *via* the TIFA pathway. (a) WT (left) or *TIFA*^−/−^ (right) HT-29 cells were left untreated (control), stimulated with ADP-H (10^−6^M), control media or *F. nucleatum* supernatant prior treatment with 5-fluoroucil (5-Fluo, 100 μM, gray bars) or without treatment (black bars). Cell viability HT-29 cell viability was monitored with MTS assay and normalized to the MTS response of untreated cells. B-E. Real-time qPCR (RT-qPCR) showing *BIRC3* (b), *TNAIP3* (c), *ICAM-1* (d), *ATG7* (e), *ULK1* (f) and *CCND1* (g) relative expression to *GAPDH* in WT (left) or *TIFA*^−/−^ (right) HT-29 cells, untreated (control), stimulated with ADP-H (10^−6^M), control media or *F. nucleatum* supernatant for 6 h. Results were normalized on *GAPDH* and expressed as 2^−ΔΔCt^ toward unstimulated cells. Data represent ≥ 3 independent experiments. Statistical significance was assessed using one-way ANOVA followed by Tukey’s multiple comparisons test. ****P < .0001; ***P < .001; **P < .01; *P < .05; *P* <.05 was considered as not significant (ns).

Recent studies have shown that *Fusobacterium* promotes CRC development and chemoresistance in a cell contact-dependent manner by targeting the innate immune receptor pathway TLR4/NF-κB. This process leads to the upregulation of miR21-dependent IEC proliferation, increased expression of the anti-apoptotic gene *BIRC3*, and activation of the autophagy pathway.^18,19,24^ Here, we aimed to investigate whether secreted ADP-H might also contribute to the oncogenic effects of *F. nucleatum*, without need for direct cell contact, through the ALPK1/TIFA-mediated activation of NF-κB. To explore this hypothesis, we assessed whether *F. nucleatum* supernatant could mitigate the cytotoxicity induced by the anti-carcinogenic drug 5-Fluoroucil (5-Fluo) in WT and TIFA^−/−^ HT-29 cells (Figure 7a). Our results showed that *F. nucleatum* supernatant reduced the cytotoxicity induced by 5-Fluo in HT-29 WT cells, albeit with a diminished effect in HT-29 TIFA^−/−^ cells. Notably, treatment with the ALPK1 ligand, ADP-H, alone did not recapitulate the effect observed with the bacterial supernatant. Furthermore, *F. nucleatum* supernatant promoted the proliferation of HT-29 WT cells but had no impact on TIFA^−/−^ cell (Figure 7a). Our data suggest that *F. nucleatum* supernatant contributes to the proliferation and chemoresistance of CRC cells *in vitro*, partially through the ALPK1-TIFA pathway.

*F. nucleatum* has been reported to promote CRC carcinogenesis by influencing various pathways, including autophagy signaling, anti-apoptotic responses, inflammation, cell growth and CRC cell adhesion.^18,19,21,34,45^ To gain further insights into the mechanisms involved in *F. nucleatum*-mediated CRC pathogenesis, we investigated the role of the ALPK1-TIFA axis in these targeted pathways. We thus assessed the impact of *F. nucleatum* supernatant on the expression of selected genes involved in autophagy (*ULK1* and *ATG7*), cell growth (CCDN1), cell adhesion (ICAM1) and anti-apoptotic responses (*BIRC3* and *TNFAIP3*). WT and TIFA^−/−^ HT-29 cells were incubated for 6 h with *F. nucleatum* supernatants, non-inoculated control medium, or ADP-H prior to RNA extraction and RT-qPCR (Figures 7b–g). Our data revealed that *ATG7* and *CCND1* expression remained unaffected by *F. nucleatum* supernatant in HT-29 cells, regardless of the genetic background. *F. nucleatum* supernatant increased *ULK1* expression in both WT and TIFA^−/−^ cells, while ADP-H failed to do so. These results confirm the published results^18^ and suggest that *F. nucleatum* activates the autophagic pathway independently of ALPK1/TIFA. In contrast, our data showed a significant TIFA-dependent upregulation of *BIRC3, TNFAIP3* and *ICAM1* expression induced by both *F. nucleatum* supernatant and ADP-H. Importantly, this enhanced transcription was TIFA-dependent (Figures 7b–d). These findings confirm the induction of *ICAM1* expression by *F. nucleatum* and highlight that it is mediated by ADP-H released by the bacterium.^34^ Altogether, our results demonstrate that *F. nucleatum* promotes the expression of anti-apoptotic and adhesion genes involved in *Fusobacterium*-mediated oncogenic mechanisms.^19,34^ This promotion occurs in a TIFA-dependent manner, underscoring the relevance of this pathway in CRC pathogenesis.

## Discussion

Colorectal cancer (CRC) is a multifactorial disease influenced by both genetic and environmental factors.^56^ Recent discoveries have placed the gut microbiota at the forefront of cancer research.^56,57^ Notably, metagenomic studies comparing the microbiomes of healthy individuals with those of CRC patients have unveiled correlations between microbiota composition and CRC outcome.^3,8,11–13,16,17^ These studies have revealed significant microbiota signatures, with over 20 bacterial species, primarily originating from the oral cavity ecosystem, associated with various aspect of CRC including its development, severity, and resistance to chemotherapy.

*Fusobacterium* is implicated in the promotion of oncogenesis through a multitude of mechanisms, including DNA damage, hyperproliferation, malignant transformation in epithelial cells, and the development of chemoresistance.^18,21,22,24^ Recent studies have highlighted the role of *Fusobacterium* in driving CRC development and enhancing chemoresistance. These effects involved the activation of the innate immune receptor pathway TLR4/NF-κB, resulting in the upregulation of miR21-dependent IEC proliferation and the expression of the anti-apoptotic gene *BIRC3* along with the activation of the autophagy pathway.^18,19,24^ These studies underscore the role of TLR4-dependent signaling pathways in various stages of carcinogenesis. However, apart from TLR4, only a limited number of studies have explored the connection between PRR and their role in carcinogenesis concerning intestinal bacteria, such as *F. nucleatum*. ^25–27^

In our study, we report that the CRC-associated bacterium *F. nucleatum* releases extracellular heptose-related metabolites capable of activating NF-κB *in vitro* through the ALPK1/TIFA signaling pathway. Importantly, this phenotype is not unique to *F. nucleatum* alone, as it is conserved among other members of the *Fusobacterium* genus associated with CRC, such as *F. varium* and *F. naviforme* (Figure 3).^58^ Our work, along with previous studies, has unveiled that both commensal and pathogenic bacteria, including *Akkermansia*, *Neisseria* and *Campylobacter* share the capacity to release extracellularly heptose-related molecules.^31,33,59^ Of note, this feature was not shared by a wide range of supernatants derived from intestinal Gram negative bacteria.^31^ Through our results, we identified butyrate, which is abundantly produced by fusobacterial species, as a synergistic molecule that enhances the NF-κB response to heptose. Our data strongly suggest that the molecule responsible for ALPK1 activation is related to heptose, specifically ADP-H. This conclusion is supported by our demonstration that the expression of *F. nucleatum* enzymes involved in LPS biogenesis, *hldA* (responsible for HBP production) or both *hldA/hldC* (for HBP production followed by its modification to ADP-H) in Δ*Hld E. coli*, is sufficient to activate NF-κB in a TIFA-dependent manner. Notably, the expression of both *hldA* and *hldC* is necessary for optimal NF-κB activation, underscoring the potency of ADP-H as the primary activator. Along the same line, ADP-H initiates rapid TIFAsome formation, whereas HBP necessitates intracellular processing by host adenyltransferases into ADP-H-7-P to achieve the same effect.^28,29^ Our assessment of TIFAsome formation in permeabilized cells 30 min after incubation of with *F. nucleatum* supernatant is a characteristic specific to the ALPK1-ligand ADP-H.^28^ Moreover, our experiments have confirmed that the *F. nucleatum* NF-κB-activating compound, like ADP-H, undergoes hydrolysis by phosphodiesterase, providing strong evidence that the released compound is indeed ADP-H.^49^

The central transcription factor NF-κB plays a pivotal role in inflammation and is associated with tumor promotion in colon cancers.^60^ Prior research has unveiled the multifaceted involvement of *F. nucleatum* in CRC development and chemoresistance, with many of these mechanisms implicating NF-κB activation. *F. nucleatum* induces the expression of IL8, a pro-inflammatory and pro-angiogenic chemokine. IL8 activation triggers the PI3K and MAPK signaling pathways, fostering an oncogenic environment that promotes cell proliferation and survival.^61,62^ Upon *F. nucleatum* infection, ALPK1 activation leads to the expression of ICAM-1, which facilitates CRC cell adhesion to endothelial cells, promoting metastasis.^34,63^ *F. nucleatum*-induced NF-κB activation leads to the upregulation of *BIRC3*, an anti-apoptotic gene involved in IEC proliferation, and its dysregulation is associated with tumor cell growth and chemoresistance.^19,64,65^ Through TLR4, *F. nucleatum* triggers the activation of *ULK1* and *ATG7*, both playing crucial roles during the initial stages of autophagy. Autophagy is a cellular pathway important for maintaining intestinal homeostasis, promoting cell survival^66^ and potentially supporting cancer development and cell chemoresistance. By activating PRR and the NF-κB response, *F. nucleatum* thus orchestrates intestinal inflammation, cell-cell adhesion, autophagy fostering an environment conducive to tumor development.

Our *in vitro* findings unveil the significance of the ALPK1-TIFA pathway as a novel contributor to the proliferation and chemoresistance of CRC cells elicited by *F. nucleatum*. We show that ADP-H, released by *Fusobacterium*, is sufficient to recapitulate the increase in *CXCL8* (IL8), *ICAM1*, *BIRC3* and *TNFAIP3* and that these effects are dependent on ALPK1/TIFA activation within IECs. Interestingly, the regulation of *ULK1* was independent of this pathway, suggesting the potential involvement of other *Fusobacterium*-released molecules in its oncogenic effects. Moreover, the activation of the ALPK1-TIFA-NF-κB axis by ADP-H has previously been directly associated with oncogenic processes in gastric cells, in promoting replication stress and DNA damage induced by *Helicobacter*. ^42^ *Fusobacterium* has also been implicated in DNA damage in various cell-types.^22,67–69^ Therefore, we can speculate that ADP-H released by *Fusobacterium* may play a role in this DNA-damaging activity.

Here, we have uncovered a previously unreported phenomenon, the released ADP-H-related molecules by *Fusobacterium* species in their microenvironment. This release is associated with pro-oncogenic effects that do not rely on direct bacterial contact with epithelial cells. Notably the activation of the ADP-H-ALPK1-TIFA axis is independent of the contact between the bacterium and IECs, raising the possibility that it might be involved in the early stages of oncogenesis. This hypothesis presents a promising avenue for further investigation.

We have previously reported that the commensal bacterium *Akkermansia muciniphila* activates the ALPK1 pathway, which participates in the maintenance of intestinal barrier functions.^31^ Our data, on both *A*. *muciniphila* and *F. nucleatum* point out to an ADP-H and ALPK1-dependent induction of *BIRC3* and *TNFAIP3* expression.^31^ Interestingly, these genes exhibit dual functions, promoting gut homeostasis by inducing IEC proliferation and crypt regeneration^64,65^ while also being associated with tumor cell growth and survival.^19,65^ This duality emphasizes the critical importance of context in determining their effects.

In conclusion, our study has demonstrated the upregulation of oncogenic genes in CRC cells due to the *F. nucleatum* microenvironment, mediated by the release of ADP-H and the activation of the ALPK1-TIFA axis. These findings underscore the relevance of this pathway in CRC pathogenesis. Our results are supported by studies that have associated *ALPK1* single-nucleotide polymorphisms (SNPs) and expression with various chronic inflammatory diseases and different cancers, including CRC.^36–40^ Interestingly, recent studies have revealed links between bacteria, including *Fusobacterium* and a wide range of solid tumors including breast, skin, and pancreatic cancers. This suggests the existence of tumor-specific microbiota.^70^ Therefore, our findings provide new insights into the role of tumor-associated bacteria in CRC, with potential application to other cancer types linked with ALPK1, such as lung, breast, and oral cancer.^71^

## Materials and methods

### Cell culture, reporter systems and CRISPR-Cas 9 deletion

HT-29 (American Type Culture Collection, ATCC) and HEK (InvivoGen) cells were grown in RPMI1640 GlutaMAX; HeLa (ATCC) in DMEM GlutaMAX medium (Gibco), both supplemented with 10% heat-inactivated fetal bovine serum (FBS, Eurobio), with 50 IU/mL penicillin, 50 μg/mL streptomycin and 10%, 100 mM Hepes, 10 mM non-essential amino acids (Gibco). HCT116 (ATCC) cells were grown in DMEM GlutaMax with 10% heat-inactivated bovine serum (Gibco) and non-essential amino acids (Gibco). Cells were grown at 37°C in a humidified atmosphere containing 5% CO_2_. HT-29, HCT116 and HEK293 cells stably expressing secreted alkaline phosphatase (SEAP, pNiflty, InvivoGen) reporter gene were used to monitor NF-κB activation (HT-29-NF-κB, HCT116-NF-κB and HEK-NF-κB).^72^ HeLa cells stably expressing TIFA-GFP were used to monitor TIFA oligomerization.^28^ CRISPR-Cas 9 deletion of *ALPK1*, *TIFA*, *MYD88* and *TRAF6* in HEK293 and HT-29 cells has been previously published.^31^

### Reagents

NOD1 inhibitor (ML130, Sigma) and NOD1 ligand (IE-DAP, InvivoGen) were used at 10 µM and 10 µg/ml, respectively. ADP-heptose (ADP-H, InvivoGen) was used at concentrations ranging from 10^−8^ to 10^−6^M.

### Culture of commensal bacteria, preparation of supernatants and short chain fatty acid (SCFA) concentration assessment

Bacterial strains were obtained from the DSMZ-German Collection and grown for 24 h in specific media and anaerobic culture conditions according to the Hungate method^73^ (Supplementary Table S1). *Fusobacterium* supernatants were harvested after centrifugation at 5,000 × g for 10 min, filtered through 0.22 μm PES filters, and stored at −80°C. Quality controls were performed using the Gram staining method, aerobic growth test, and fresh observations under a microscope. The concentrations of SCFAs produced by cultured bacteria were assessed by gas chromatography as described in.^51^

### Cell stimulation and reporter system assays

For each experiment, cells were seeded at 3.10^4^ cells per well in 96-well plates for 24–48 h and then stimulated for 24 h with bacterial supernatants or non-inoculated bacterial culture medium as a control in a total culture volume of 100 μL per well prior to the reporter assay. When indicated, the cells were incubated with ML130 (10 µM), IE-DAP (10 µg/ml), or ADP-H (10^−8^ to 10^−6^M). SEAP was revealed with the Quanti-Blue reagent (InvivoGen) using a microplate reader (655 nm Infinite 200, Tecan). NF-κB activation was normalized to the control, that is, unstimulated cells. Experiments were performed in triplicate for at least three independent biological assays.

### CIP and PDE treatments of F. nucleatum supernatant

*F. nucleatum* supernatants were treated with calf intestinal alkaline phosphatase (CIP, 100 U/ml) or phosphodiesterase (PDE, 30 mU/ml) from *Crotalus adamanteus* for 30 min, at 37°C as published followed by a 3 kDa-sieved filter step to eliminate the enzymes.^31,49^ The control media were treated similarly. The cells were stimulated with the treated supernatants for 24 h, and the NF-κB signal was monitored.

### Cloning and expression of F. nucleatum hldA and hldC into E.Coli ΔHldE

*E. coli* K-12 WT and *ΔHldE E. coli* (JW3024) were obtained from Keio collection^74^ (Dharmacon). *hldA* from *F. n. nucleatum* was amplified using the following primers: (i) FhldAfor_HindIII (5’-GGGGAAGCTTAGGAGGTAAATA*ATG*ATAAGTAAATTAATAG-3’), creating a new HindIII restriction site (AAGCTT) and adding a Shine-Dalgarno sequence (AGGAGG) located six bases upstream of the start codon *ATG* and (ii) FhldArev_BamHI (5’-GGGGGGATCCTTAATTATTACTATATATACTGTTA-3’), creating a new BamHI restriction site (GGATCC). The 998 pb fragment was amplified using Phusion High-Fidelity DNA polymerase, A-tail with GoTaq polymerase, and cloned into the pGEM-T Easy vector (Promega Corporation) to generate pGEM-T-FhldA. After the HindIII BamHI restriction of pGEM-T-FhldA, the FhldA fragment was cloned into pGBM6 to generate the pGB-hldA vector (spc/str). Similarly, *hldC* from *F. n. nucleatum* was cloned into pBAD24 generated pBAD-FhldC vector (amp) using the following primers: (i) FhldCfor_NheI (5’-GGGGGCTAGCAGGAGGTAAATA*ATG*GAAAGGTGGGTTTTTA-3’) and (ii) FhldCrev_HindIII (5’-GGGGAAGCTTCTATTTTTTATTAATTTTTTC-3’). At each cloning step, the insertion and fragment were verified by sequencing.

Both pGB-hldA, pBAD-hldC, and the empty vectors pBAD24 and pGBM6 were purified and electroporated into *E. coli* Δ*HldE*. Overnight bacterial cultures were washed in PBS, resuspended at OD = 1, boiled for 30 min, and stored at −20°C until use.

### Real-time qPCR

HT-29 or HEK cells were seeded in 6-well culture plates at densities of 1.10^6^ per well 24 h before stimulation. HCT116 were seeded in 12-well plates at 0.8.10^6^ cells per well 24 h prior stimulation. Total RNA was extracted using the RNeasy mini-Kit (Qiagen) according to the manufacturer’s protocols with DNase I treatment (R&D Systems). cDNA was synthesized from 2 µg of RNA using the High-Capacity cDNA Reverse Transcription Kit (Applied Biosystems), and 100 ng was used to conduct qPCRs on ABI Prism 7700 (Applied Biosystems). The following Taqman Gene expression assay probes were used: *GAPDH* Hs02758991_g1, *ALPK1* Hs01567926, *TIFA* Hs00385268, *ATG7* Hs00893766, *ULK1* Hs00177504, *BIRC3* Hs00985029, *TNFAIP3* Hs00234713, *CXCL8* Hs00174103_m1. *GAPDH* was used for normalization. Comparisons were done with 2^−ΔΔCt^. Samples were tested in duplicates, at least in biological triplicates.

### siRNA assay

HeLa cells seeded in 96-well plates (8000 cells/well) were transfected with 20 nM siRNA as previously described.^30^ The cells were transfected with a validated ALPK1 siRNA (s37074, Ambion) and a non-targeting sequence (4390843, Ambion). For the ALPK1 complementation experiment, 48 h after siRNA transfection, cells were transfected with an *ALPK1* cDNA construct (pCMV-ALPK1) or an empty vector (pCMV) using FuGENE 6 (Roche) prior to TIFAsome analysis, as previously described.^28^

### TIFAsome assay and quantification

HeLa TIFA-GFP cells were seeded in 96-well plates for at least one day before the experiment. Cells were washed in permeabilization buffer as previously published.^28^ Cells were then incubated with digitonin alone as a control, digitonin plus HBP, ADP-H, medium control, or bacterial supernatant for 30 min in permeabilization buffer. To monitor TIFA oligomerization, cells were fixed after 30 min of stimulation with 4% PFA. Images were acquired using ImageXpress Micro (Molecular Devices). Image analysis and TIFAsomes quantification were performed using the custom module editor of MetaXpress as previously published.^28^

### ELISA

HT-29 cells were seeded at 1 × 10^6^ cells per well in six well-plates 24 h prior to incubation with bacterial supernatants, non-inoculated bacterial media, or ADP-H. Supernatants were collected 24 h later and assessed by ELISA following the manufacturer’s instructions using the IL8 ELISA kit (BioLegend).

### Cell proliferation assay

The percentage of viable cells was determined by MTS measurement using CellTiter 96 Aqueous One solution (Promega) according to the manufacturer’s recommendations. Briefly, the reagent solution was directly added to the culture wells, and the plates were incubated 0,5 for 1 h before measuring the absorbance at 490 nm using a microplate reader (655 nm Infinite 200, Tecan). Experiments were performed in technical triplicates for at least three independent biological assays.

### Statistical analysis

All experiments were conducted in technical triplicates for at least three independent biological assays (otherwise specified), and the results are expressed as mean ± SD fold change toward the control condition. Data were represented and analyzed using GraphPad Prism (GraphPad Software). Differences between individual groups were verified using Mann-Whitney test or one-way ANOVA followed by Tukey’s multiple comparisons test. P-values are indicated as follows: ****P <.0001; ***P < .001; **P < .01; *P < .05; *P* >.05 was considered not significant (ns).

## Supplementary Material

Supplemental Material

Supplementary figS2 resub.tiff

Supplementary fig S1 resub.tiff

Supplementary Table S1.xlsx

## Data Availability

All data are contained in the article and the supporting information.
