# Supplementary material for: Fusobacterium nucleatum promotes inflammatory and anti-apoptotic responses in colorectal cancer cells via ADP-heptose release and ALPK1/TIFA axis activation
Source: Gut Microbes. 2023 Dec 21;16(1):2295384. doi: 10.1080/19490976.2023.2295384 (PMC10761154; doi:10.1080/19490976.2023.2295384)
Supplement: Supplemental Material [file KGMI_A_2295384_SM8905.docx]

Supplementary Figure S1: *F. nucleatum* supernatant and ADP-H activate NF-κB and *CXCL8* expression in HCT116 cell-line. A. HCT116 NF-κB-reporter cells were stimulated with ADP-H, *F. nucleatum* supernatant or control media for 24 h. NF-κB activation was measured by SEAP secretion and expressed as mean ± SD fold change towards unstimulated cells. B. *CXCL8* relative expression to GAPDH in HCT116 stimulated with ADP-H (10^-6^M), *F. nucleatum* supernatant or control media for 6 h expressed as 2^-ΔΔCt^ towards unstimulated cells. Data analysis: one-way ANOVA followed by Tukey’s multiple comparisons test was used, ****P<0,0001; ***P<0,001; **P<0,01; *P<0,05; P<0.05 was considered as not significant (ns).

Supplementary Figure S2: Alignment of *E. coli* K12 *HldE* and *F. nucleatum HldA* and HldC amino acid sequences. Dark and gray shading indicates conserved and positive residues, respectively.
