# Supplementary figures and images for: Fusobacterium nucleatum promotes inflammatory and anti-apoptotic responses in colorectal cancer cells via ADP-heptose release and ALPK1/TIFA axis activation
Source: Gut Microbes. 2023 Dec 21;16(1):2295384. doi: 10.1080/19490976.2023.2295384 (PMC10761154; doi:10.1080/19490976.2023.2295384)

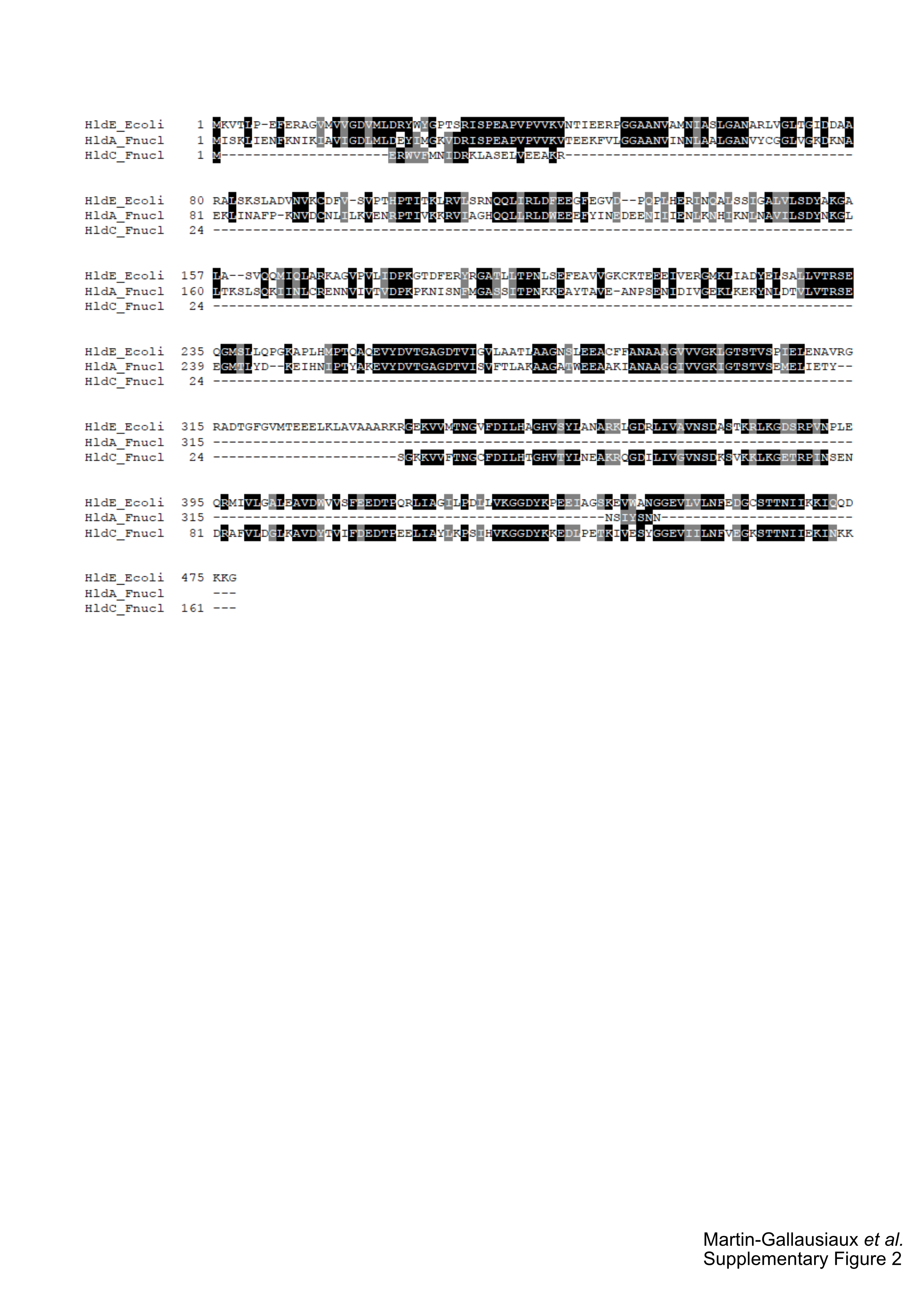

Supplement: Supplementary figS2 resub.tiff [file KGMI_A_2295384_SM1666.tiff]

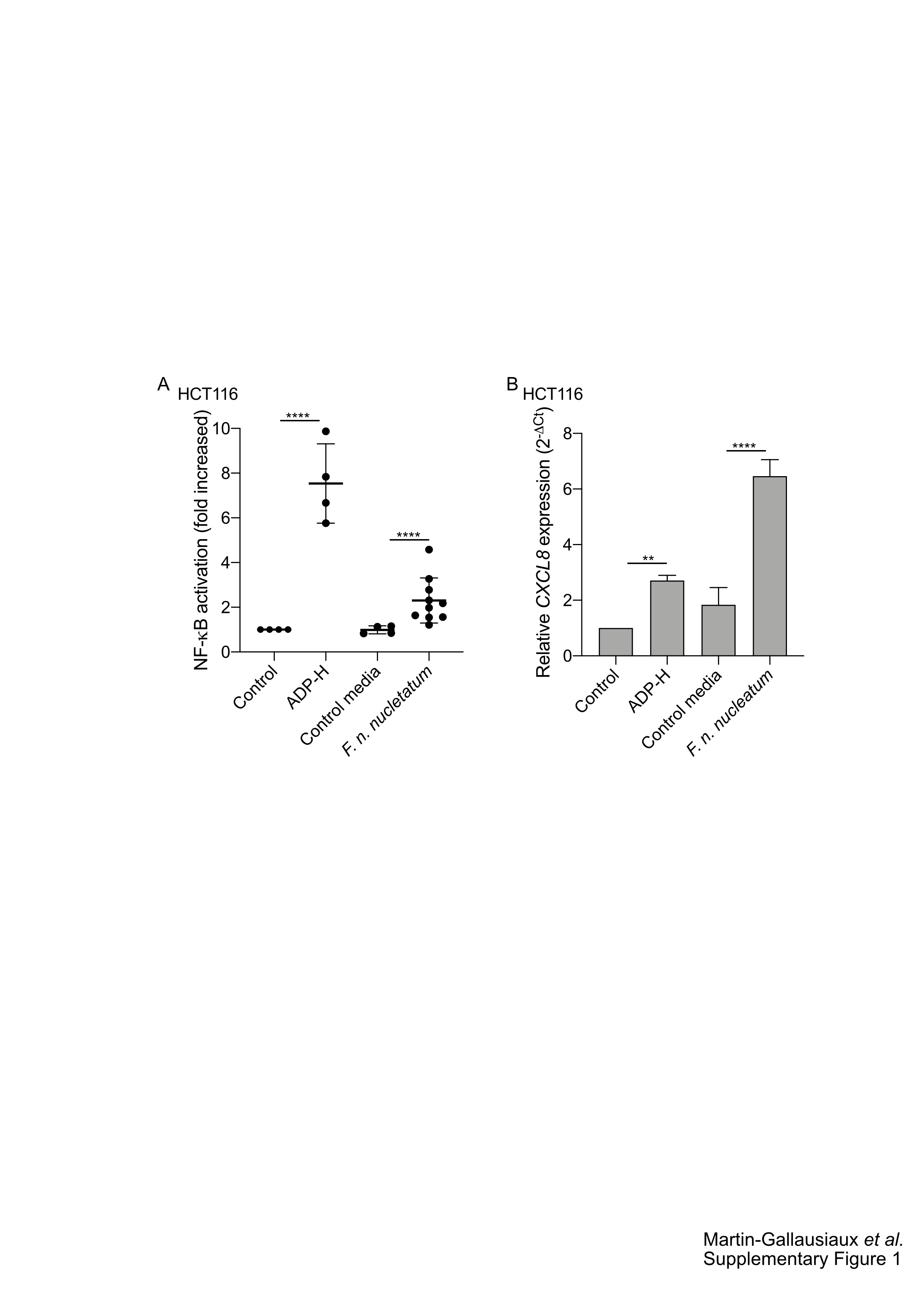

Supplement: Supplementary fig S1 resub.tiff [file KGMI_A_2295384_SM1665.tiff]
